# Exploring the molecular mechanisms of electron shuttling across the microbe/metal space

**DOI:** 10.3389/fmicb.2014.00318

**Published:** 2014-06-27

**Authors:** Catarina M. Paquete, Bruno M. Fonseca, Davide R. Cruz, Tiago M. Pereira, Isabel Pacheco, Cláudio M. Soares, Ricardo O. Louro

**Affiliations:** Instituto de Tecnologia Química e Biológica António Xavier, Universidade Nova de LisboaOeiras, Portugal

**Keywords:** electron shuttles, *Shewanella*, outer membrane cytochromes, mediated electron transfer, extracellular respiration

## Abstract

Dissimilatory metal reducing organisms play key roles in the biogeochemical cycle of metals as well as in the durability of submerged and buried metallic structures. The molecular mechanisms that support electron transfer across the microbe-metal interface in these organisms remain poorly explored. It is known that outer membrane proteins, in particular multiheme cytochromes, are essential for this type of metabolism, being responsible for direct and indirect, via electron shuttles, interaction with the insoluble electron acceptors. Soluble electron shuttles such as flavins, phenazines, and humic acids are known to enhance extracellular electron transfer. In this work, this phenomenon was explored. All known outer membrane decaheme cytochromes from *Shewanella oneidensis* MR-1 with known metal terminal reductase activity and a undecaheme cytochrome from *Shewanella* sp. HRCR-6 were expressed and purified. Their interactions with soluble electron shuttles were studied using stopped-flow kinetics, NMR spectroscopy, and molecular simulations. The results show that despite the structural similarities, expected from the available structural data and sequence homology, the detailed characteristics of their interactions with soluble electron shuttles are different. MtrC and OmcA appear to interact with a variety of different electron shuttles in the close vicinity of some of their hemes, and with affinities that are biologically relevant for the concentrations typical found in the medium for this type of compounds. All data support a view of a distant interaction between the hemes of MtrF and the electron shuttles. For UndA a clear structural characterization was achieved for the interaction with AQDS a humic acid analog. These results provide guidance for future work of the manipulation of these proteins toward modulation of their role in metal attachment and reduction.

## Introduction

Submerged and underground metallic structures are subject to corrosion pressures that can negatively impact essential infrastructure of industrial activities such as water distribution and oil and gas exploration. Oxygen, even in the ppm concentration range, is the major source of corrosion of iron based structures such as steel pipes. In damp environments, in addition to abiotic factors, microorganisms exert a strong influence in the durability of these structures (Popoola et al., 2013).

The view of the interaction between anaerobic organisms and submerged metallic structures has historically been dominated by the role of their metabolism in processes of biocorrosion (Dubiel et al., 2002). In particular, sulfate reducing bacteria have been a notorious focus of attention of the oil and gas industry due to their role in field souring and pit corrosion (Hamilton, 1998). A considerable investment has been made to identify efficient strategies to control their presence such as injection of nitrate and nitrite (Schwermer et al., 2008; Voordouw, 2011). Notwithstanding, it was shown that biofilms of dissimilatory metal-reducing bacteria (DMRB) are capable of conferring bioprotection by limiting the access of dissolved oxygen to the metal and discharging intracellular electrons generated from the degradation of organic compounds to solid metallic structures or minerals outside the cell (Dubiel et al., 2002). This metabolic activity is termed extracellular electron transfer, and has contributed significantly for the biogeochemical cycling of metals on Earth (Fredrickson and Zachara, 2008). In addition to the geobiological interest of this process, numerous potential biotechnological applications have recently emerged that take advantage of this phenomenon, such as microbial fuel cells (MFC), or the bioremediation of aquifers or sediments contaminated with heavy metals or radionuclides (Fredrickson et al., 2008; Logan, 2009; Lovley, 2012). For biofilms that are thicker than a cell monolayer, indirect electron transfer to the metal substrate is essential to sustain the viability for those cells not directly attached to the metal surface. Gram-negative gama-proteobacteria are known members of communities in marine biofilms attached to metallic structures (Bermont-Bouis et al., 2007). One of the best-studied DMRB that has gained the status of model organism is the facultative anaerobe Gram-negative gama-proteobacterium *Shewanella oneidensis* strain MR-1 (SOMR1). This was the first dissimilatory metal reducing organism reported in the literature (Myers and Nealson, 1988). Its genome codes for 41 putative *c*-type cytochromes, many of which are associated with the membranes (Meyer et al., 2004; Romine et al., 2008). Several of these cytochromes participate in metal reducing (Mtr) extracellular electron transfer and this process relies on the so-called porin-cytochrome complexes that span the outer membrane (Richardson et al., 2012). These multiprotein complexes provide the molecular infrastructure that allows the conduction of electrons from the periplasmic cytochromes to the cell-surface terminal reductases (Richardson et al., 2012). In SOMR1, site directed mutagenesis studies showed that three of these transmembrane redox supercomplexes, MtrCAB-OmcA, MtrDEF, and DmsABCED have an experimentally identifiable physiological role (Bretschger et al., 2007; Coursolle and Gralnick, 2012). The first two were assigned to metal reduction metabolism whereas the third was dedicated to the reduction of DMSO (Gralnick et al., 2006). In MtrCAB-OmcA complex the decaheme *c*-type cytochromes MtrC and OmcA are the putative extracellular terminal metal reductases. Of these two, only OmcA has been structurally characterized at low resolution using SAXS (Johs et al., 2010). In the MtrDEF complex, the decaheme *c*-type cytochrome MtrF is the putative terminal metal reductase and its three-dimensional structure was solved by X-ray crystallography (pdb code: 3PMQ) (Clarke et al., 2011). These three proteins reveal a significant degree of sequence homology among them and also with another cytochrome called UndA that contains eleven *c*-type hemes. This is a putative metal terminal reductase found in a closely related *Shewanella* species isolated from an uranium contaminated river for which the high resolution structure has also been reported (pdb code: 3UCP) (Edwards et al., 2012b). All of these metal terminal reductases perform their physiological activity by establishing direct contact with mineral surfaces (Ross et al., 2009) and also indirectly using electron shuttles of low molecular weight (Lies et al., 2005; Marsili et al., 2008; Von Canstein et al., 2008). Mutants lacking these terminal reductases showed strong growth deficiency under dissimilatory metal-reducing conditions. MtrC is responsible for most of the electron transfer and OmcA combines this activity with a role in cellular attachment to solid surfaces (Coursolle and Gralnick, 2010). Interestingly, both OmcA and MtrF can compensate for the loss of MtrC in the reduction of several electron acceptors (Myers and Myers, 2001; Bücking et al., 2010; Coursolle and Gralnick, 2010, 2012), which can be a consequence of the structural homology among these proteins (Edwards et al., 2012a,b). For example, under manganese-reducing conditions, OmcA can deliver electrons to the terminal acceptor in the absence of MtrC (Myers and Myers, 2001; Bücking et al., 2010), while in iron-reducing conditions MtrF can substitute for MtrC giving origin to a functional MtrABF complex (Coursolle and Gralnick, 2010, 2012).

The participation of the outer membrane multiheme cytochromes in the direct and indirect extracellular electron transfer appears however to be strictly regulated by environmental conditions. When SOMR1 and also *Shewanella iolytica* PV-4 were grown using poised potential electrodes, direct electron transfer occurred for potentials more positive than those assigned to the outer membrane cytochromes whereas indirect transfer occurred for more negative potentials (Baron et al., 2009; Liu et al., 2010).

**Figure 1:**
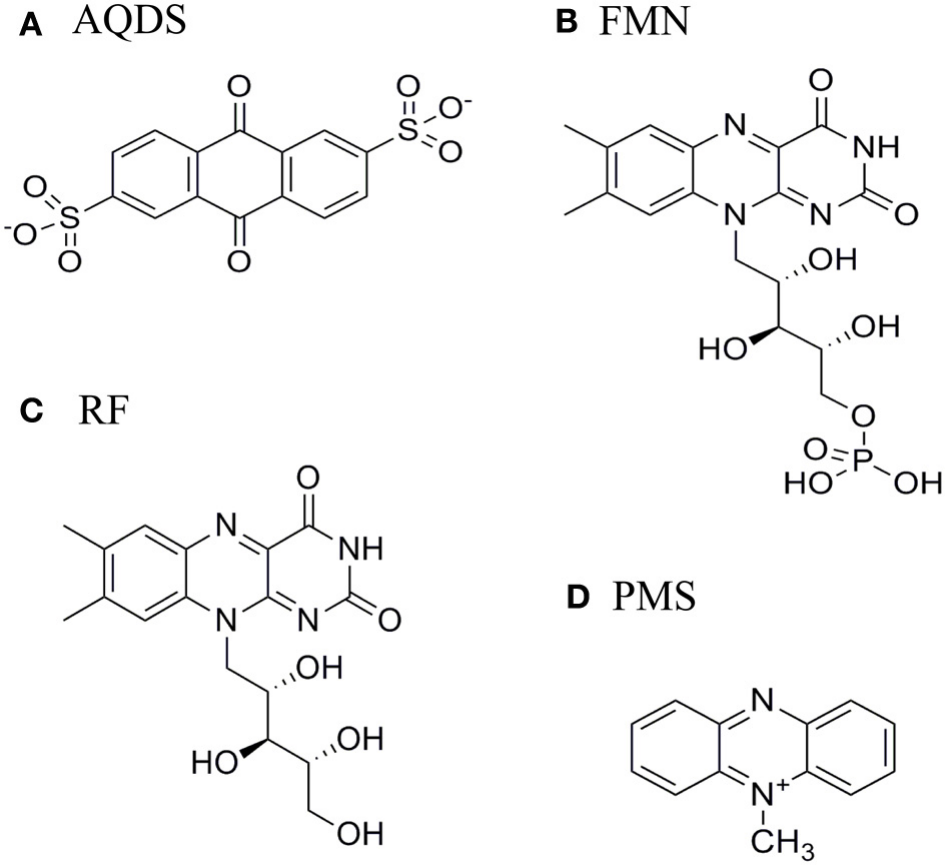
**Structure of (A) anthraquinone-2,6-disulphonate (AQDS), (B) Flavin mononucleotide (FMN), (C) Riboflavin (RF), and (D) Phenazine methosulfate (PMS)**.

Indirect extracellular electron transfer to metals relies on redox active organic compounds of low molecular weight that stimulate the reduction of metals and inert electrodes by acting as electron shuttles between microbes and conductive solids (Newman and Kolter, 2000; Hernandez et al., 2004; Lovley, 2006). These compounds can be endogenously produced or scavenged from the medium. Early reports showed that quinone-containing humic substances promote the dissimilatory reduction of iron (Lovley et al., 1996), while more recent studies have revealed that flavins accelerate the reduction of both iron oxide minerals and electrodes (Von Canstein et al., 2008; Ross et al., 2009; Coursolle et al., 2010). Both riboflavin (RF) and flavin mononucleotide (FMN) were observed to accumulate in supernatants of SOMR1 grown under anaerobic and aerobic conditions with concentrations up to the micromolar range (Marsili et al., 2008; Von Canstein et al., 2008; Ross et al., 2009). Phenazines and redox-active antibiotics were also shown to function as electron carriers to reduce minerals by SOMR1 (Hernandez et al., 2004). Interestingly, all these molecules have redox-active functional groups that are based on multiple aromatic rings arranged linearly (Figure 1).

It was proposed that the small electron shuttles are reduced microbiologically and subsequently reoxidized by poorly crystalline iron minerals. Nevertheless the molecular basis of the interaction between the electron shuttle and the microbe remains to be clarified. Flavins were shown to accelerate the reduction of iron oxides and increase current production from electrode-grown culture by directly interacting with the outer membrane cytochromes (Marsili et al., 2008; Ross et al., 2009; Coursolle et al., 2010; Okamoto et al., 2013). However, the affinity and stoichiometry of these interactions as well as the binding site in the outer membrane cytochromes for which the structure is known remains unrevealed (Clarke et al., 2011; Edwards et al., 2012b).

Recently the complex network of redox interactions across the periplasmic space of SOMR-1 was described shedding light on the details of electron transfer from the cytoplasmic membrane to the outer membrane terminal metal oxidoreductases (Fonseca et al., 2013). To extend the detailed characterization of extracellular respiration to the events taking place across the space between cells and metallic surfaces or minerals, electron transfer kinetic experiments and binding experiments followed by nuclear magnetic resonance spectroscopy (NMR) were used to obtain molecular insights on the interaction between the outer membrane cytochromes of *Shewanella* and soluble electron shuttles. Flavins, a phenazine and anthraquinone 2,6-disulfonate (AQDS), an humic substance analog, were selected given their physiological relevance and different chemical nature. This allows for the first time the molecular characterization of the interaction between the terminal oxidoreductases and mobile electron shuttles. The results were complemented with molecular docking simulations to identify the likely candidate locations of interaction between the outer membrane cytochromes and electron shuttles.

## Materials and methods

### Protein production

#### Bacterial strains and growth conditions

The cloning vectors (pBAD202/D-TOPO) containing the MtrC, MtrF, OmcA, and UndA genes were constructed accordingly to the procedure available in the literature (Shi et al., 2005) to insert a His_6_ tag at the C-terminus of the expressed proteins. These plasmids, kindly provided by Dr Liang Shi from the Pacific Northwest National Laboratory (Richland, WA, USA), were mutated to insert a stop codon after the gene sequence, allowing the removal of the V5 epitope and the His-tag sequence included at the C-terminus of the proteins. The lack of these sequences yielded higher levels of expression when compared with the original plasmid (unpublished results), and eliminated concerns about proper folding of the protein in the presence of the His_6_ tag at the C-terminus.

The mutation was performed for each plasmid, using a site-directed mutagenesis protocol (NZYMutagenesis kit; NZYTech, Lda.) with the primers presented in Table 1.

**Table 1:** **Primers used in this study to insert a stop codon in the expression plasmids**.

**Primer**	**Sequence (5′ → 3′)**
MtrC_Stop_For	CACACTAAAGTGAAAATGTAAGGCGAGCTCAAGCTTGAAGGT
MtrC_Stop_Rev	ACCTTCAAGCTTGAGCTCGCCTTACATTTTCACTTTAGTGTG
MtrF_Stop_For	CGCCGACGTACTCAAAGTCCATCCAATAAACTAAGGCGAGCTCAAGCTTGAAGGTAAGCC
MtrF_Stop_Rev	GGCTTACCTTCAAGCTTGAGCTCGCCTTAGTTTATTGGATGGACTTTGAGTACGTCGGCG
OmcA_Stop_For	CGCAATTGATGGAAGCACACGGTAACTAAGGCGAGCTCAAGCTTGAAGGTAAGCC
OmcA_Stop_Rev	GGCTTACCTTCAAGCTTGAGCTCGCCTTAGTTACCGTGTGCTTCCATCAATTGCG
UndA_Stop_For	GGTGTGGATAAAGTTCACCCAGTGAAGTACTAAGGCGAGCTCAAGCTTGAAGGTAAGCC
UndA_Stop_Rev	GGCTTACCTTCAAGCTTGAGCTCGCCTTAGTACTTCACTGGGTGAACTTTATCCACACC

SOMR1 was used to overexpress the recombinant outer membrane multiheme cytochromes MtrF and OmcA, taking advantage of the host cell's own mechanism for heme insertion. The SOMR1 strains were grown aerobically at 30°C in Terrific Broth (TB) medium containing 50 μg/mL kanamycin. Protein expression was induced by addition of 1 mM arabinose at the mid-log phase of growth and the cells were allowed to grow for an additional 18 h.

*Escherichia coli* JM109 (DE3) co-transformed with plasmid pEC86, containing the *ccmABCDEFGH* (cytochrome *c* maturation) genes (Arslan et al., 1998), was used to overexpress the outer membrane multiheme cytochromes MtrC and UndA. The *E. coli* strains were grown aerobically at 37°C in TB medium containing 50 μg/mL kanamycin and 35 μg/mL chloramphenicol. Protein expression was induced by addition of 1 mM arabinose at the mid-log phase of growth. The temperature was lowered to 30°C and the cells were allowed to grow for an extra 18 h.

Bacterial cells were harvested by centrifugation at 10,000 g for 10 min at 4°C.

#### Protein purification

The cell pellets from the SOMR1 cultures were resuspended in 20 mM Tris/HCl (pH 7.6) containing a protease inhibitor cocktail (Roche) and DNase I (Sigma). The disruption of the cells was attained by two passages through a French Press at 1000 psi. The crude extract was centrifuged at 200,000 g for 1 h at 4°C to remove membranes and cell debris. The supernatant containing the soluble protein fraction was used to purify the target proteins.

The cell pellets from the *E. coli* growths were resuspended in an extraction solution (0.5 M sucrose, 0.2 M Tris-HCl, 0.5 mM EDTA, 100 mg L^−1^ lysozyme, pH 7.6) and incubated on ice for 30 min with gentle stirring. The resulting spheroplasts were pelleted, and the supernatant, containing the periplasm, was cleared by ultracentrifugation at 200,000 g for 30 min at 4°C and was used to purify the target proteins.

For each target protein, the respective supernatant was dialyzed overnight against 20 mM Tris-HCl (pH 7.6) and concentrated in an ultrafiltration cell with a 30 kDa cut-off membrane. The resulting fraction was loaded on to a diethylaminoethyl (DEAE) column (GE Healthcare) previously equilibrated with 20 mM Tris/HCl (pH 7.6) and a salt gradient from 0 to 1 M NaCl in the same buffer. The fractions containing MtrF and UndA were eluted at 150 mM NaCl, while fractions containing OmcA and MtrC were eluted at 100 mM and 200 mM NaCl, respectively. Each fraction was concentrated, dialyzed and loaded separately on to a HTP (hydroxyapatite) column (Bio-Rad Laboratories), previously equilibrated with 20 mM potassium phosphate buffer (pH 7.6). In all cases, fractions containing the multiheme cytochromes MtrF, UndA, OmcA, and MtrC did not bind to the column and were eluted in the washout volume. The fractions containing the target proteins were concentrated and a final purification step was performed on a Superdex 75 column (GE Healthcare), equilibrated with 20 mM potassium phosphate buffer (pH 7.6) with 100 mM KCl, prior to use.

The chromatographic fractions were analyzed by SDS/PAGE (12% gel) and UV-visible spectroscopy to select those that contain the protein of interest. The purity of the target proteins was revealed as a single band in the gel and the protein fractions had a typical absorbance ratio A_Soret Peak_/A_280nm_ larger than 5.0.

### Kinetic experiments

#### Sample preparation

Dilutions of the proteins to the desired concentration were made from stock solutions of MtrC, MtrF, OmcA, and UndA in degassed 20 mM potassium phosphate buffer (pH 7.6) with 100 mM KCl. The actual concentration of the protein was determined by UV-visible spectroscopy using ε_552nm_ of 30,000 M^−1^cm^−1^ per heme for the reduced state of the protein (Massey, 1959).

Stock solutions of the electron shuttles AQDS, FMN, RF, and phenazine methosulfate (PMS) were prepared by the addition of weighted amounts of solid to 20 mM potassium phosphate buffer (pH 7.6) with 100 mM KCl. Dilutions of the electron shuttles were prepared in degassed buffer, and the actual concentrations were determined by UV-visible spectroscopy using ε_326nm_ = 5200 M^−1^cm^−1^ for AQDS (Shi et al., 2012), ε_445nm_ = 12,200 M^−1^cm^−1^ for FMN (Aliverti et al., 1999), ε_445nm_ = 12,500 M^−1^cm^−1^ for RF (Whitby, 1953), and ε_387nm_ = 26,300 M^−1^cm^−1^ for PMS (Dawson, 1987).

Reduced solutions of MtrC, MtrF, OmcA, and UndA were prepared by the addition of small volumes of concentrated sodium dithionite solution. To confirm that there is no excess of reducing agent, UV-visible spectra were obtained for the reduced protein using ε_314nm_ = 8000 M^−1^cm^−1^ (Dixon, 1971).

All manipulations of the protein samples and electron shuttle solutions used for the kinetic experiments were performed inside the anaerobic chamber containing less than 5 ppm of oxygen.

#### Data acquisition

The kinetic experiments were performed using a stopped-flow spectrometer (HI-TECH Scientific SF-61 DX2) placed inside an anaerobic chamber (M-Braun 150). Data were collected by measuring the light absorption changes at 552 nm. The temperature during the kinetic experiment was kept at 25°C using an external circulating bath.

For each protein, the reference values for the optical density in the fully oxidized and reduced state were obtained by mixing the protein with potassium ferricyanide and sodium dithionite, respectively. These values were used to normalize the kinetic data obtained for each protein with different electron shuttles to have reduced fraction vs. time.

### NMR experiments

#### Sample preparation and titrations

Stock solutions of the electron shuttles FMN, RF, AQDS, and PMS were prepared in ^2^H_2_O (99.9 atom %) and contained 20 mM potassium phosphate buffer (pH 7.6) with 100 mM KCl. The final concentration of these solutions was 5 mM with the exception of the RF solution which had a concentration of 200 μM.

The protein samples were lyophilized twice using ^2^H_2_O (99.9 atom %). NMR spectra obtained before and after the lyophilization were identical, showing that the protein structure was not affected by this procedure. All protein samples were dissolved in ^2^H_2_O (99.9 atom %) and contained 20 mM potassium phosphate buffer (pH 7.6) with 100 mM KCl. The concentration of the cytochrome stock samples was 500 μM each. All dilutions of the stock solutions were performed using a 20 mM potassium phosphate buffer (pH 7.6) prepared in ^2^H_2_O (99.9 atom %) with 100 mM KCl.

NMR experiments were performed at 25°C on a Bruker Avance II 500 MHz NMR spectrometer equipped with a TXI probe for ^1^H detection or a SEX probe for ^31^P detection.

NMR samples containing 50 μM of the target cytochrome were titrated against increasing concentrations of the different electron shuttles. ^1^H-1D-NMR spectra were recorded for each addition. The inverse procedure, using a sample with 50 μM of the electron shuttle to which increasing amounts of cytochrome were added, was also performed.

Because of spectral crowding in the proton frequency, advantage was taken of the existence of a single phosphorus nucleus in the case of the electron shuttle FMN. Samples containing 100 μM of FMN were prepared and titrated against increasing concentrations of the target cytochrome.^31^P-1D-NMR spectra were recorded for each addition.

The pH of the samples was measured before and after each series of additions to confirm that the pH of the solution remained unchanged. After each experiment electron shuttles were removed from the protein solutions using PD-10 desalting columns (GE Healthcare).

#### Data analysis and binding affinities

The NMR spectra were processed and analyzed using the Bruker TopSpin 2.1 software. Chemical shifts are reported in parts per million (ppm) and the spectra were calibrated using the water or the inorganic phosphate signal as internal references, in the case of the proton or phosphorus spectra, respectively.

In order to determine binding affinities between the outer membrane cytochromes and the electron shuttles, the chemical shift perturbations (Δδ_bind_) of the ^31^P NMR signal from FMN (L) resulting from the complex formation with the cytochrome (C) were plotted against the molar ratio (R) of [C]/[L]. The data were fitted with least squares minimization to the following binding model (C + nL ⇄ CL_*n*_) using an equation for fast exchange regime which corrects for dilution effects (Worrall et al., 2003):

(1a) $$\Delta \delta_{\text{bind}}=\frac{1}{2}\Delta \delta_{\text{bind}}^{\infty }\left(\text{A}-\sqrt{\left({\text{A}}^{2}-\text{4nR}\right)}\right)$$

(1b) $$\text{ A}=1+\text{nR}+\frac{\beta_{\text{d}}({\left[\text{L}\right]}_{0}\text{R +}{\left[\text{C}\right]}_{0})}{{\left[\text{L}\right]}_{0}{\left[\text{C}\right]}_{0}}$$

Where Δδ^∞^_bind_ is the maximal chemical shift perturbation of the NMR signal resulting from the complex formation between FMN and multiheme cytochrome and β_*d*_ is the macroscopic dissociation constant. [L]_0_ is the initial concentration of FMN, [C]_0_ is the stock concentration of the cytochrome, and n is the number of binding sites. Experimental uncertainty was estimated from the spectral resolution of the NMR acquisition. Only chemical shift perturbations larger than 0.025 ppm were considered significant (Díaz-Moreno et al., 2005).

### Molecular docking simulation

Molecular docking was performed using Autodock 4 (Morris et al., 1998, 2009; Huey et al., 2007). The pdb codes for the undecaheme cytochrome UndA from *Shewanella* sp. strain HRCR-6, and the decaheme cytochrome MtrF from *Shewanella oneidensis*, used in the docking experiments, were 3UCP and 3PMQ, respectively. The normal parameterization of Autodock 4 was used, considering only polar hydrogens and setting Gasteiger-Marsili partial charges (Gasteiger and Marsili, 1980) for the AQDS ligand and for the proteins. Iron atoms in all heme groups were considered to be in the oxidized state (Fe(III)). Divalent ions found at the surface of the protein and all water molecules were removed. For the UndA cytochrome, two separate docking experiments were performed, in the presence or absence of a Ca^2+^ cation. This Ca^2+^ cation site, contrary to the Mg^2+^ sites found in the same protein, seems to be functional, given that it is highly organized, with the cation surrounded by three carboxyl groups, one from a propionate side chain of the heme II group and the other two from the Asp293 and Glu295 residues. Additionally, the crystallization solutions did not contain added calcium salts (but contained added magnesium salts). The grids used for the docking experiments were sized to contain the whole protein, in order to map the exposed surface, and the grid spacing used was 0.375 Å. Each run of the genetic algorithm had 3 × 10^4^ generations, with 5 × 10^6^ energy evaluations. This process was performed 1000 times to obtain a solid clustering analysis.

## Results

### Kinetic experiments

The kinetics of oxidation of the cytochromes by the electron shuttles were measured at a 552 nm where the hemes display an intense change in absorption linked to the redox transition. The kinetic experiments report only the oxidation of the hemes of the cytochromes, since the electron shuttles used in this work do not have redox linked absorbance changes at this wavelength (Table 2).

**Table 2:** **Physicochemical properties of AQDS, FMN, RF, and PMS**.

**Chemical name**	**Reduction potential**		**ε (M^−1^ cm^−1^) [λ_max_ (nm)]**	
	**E°′ (mV)**	**References**	**Oxidized form**	**References**
Anthraquinone 2,6-disulfonate (AQDS)	−185	Shi et al., 2012	5200 [326 nm]	Shi et al., 2012
Flavin mononucleotide (FMN)	−216	Shi et al., 2012	12,200 [445 nm]	Aliverti et al., 1999
Riboflavin (RF)	−208	Shi et al., 2012	12,500 [445 nm]	Whitby, 1953
Phenazine methosulfate (PMS)	+80	Prince et al., 1981	26,300 [387 nm]	Dawson, 1987

**Figure 2:**
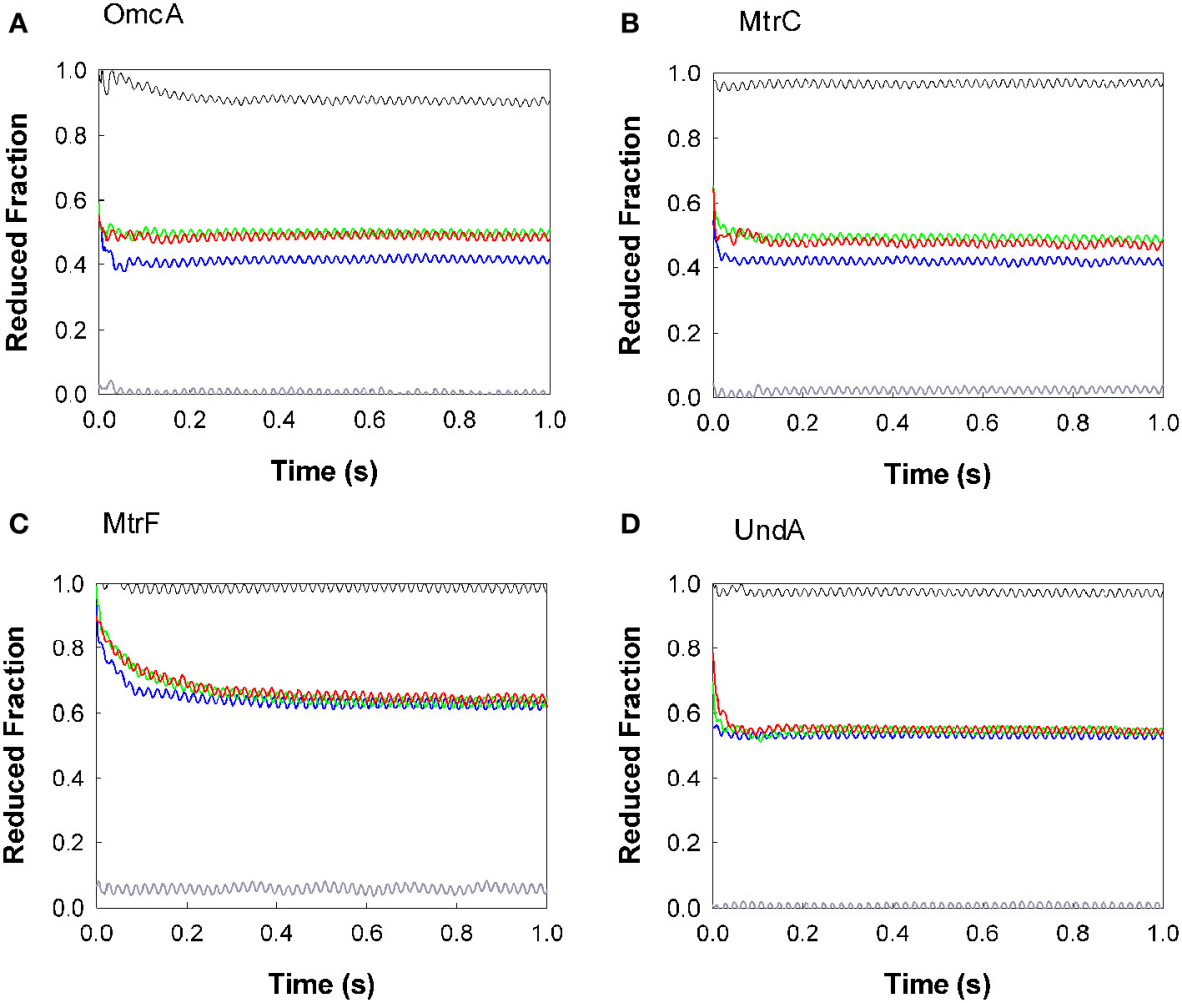
**Kinetics of oxidation of the outer membrane cytochromes by AQDS (blue), FMN (green), RF (red), and PMS (gray)**. The protein concentrations were 0.36, 0.31, 0.30, and 0.32 μM for OmcA **(A)**, MtrF **(C)**, MtrC **(B)**, and UndA **(D)** respectively. The concentration of the electron shuttles were 21 μM for AQDS, 16 μM for FMN, 11 μM for RF, and 11 μM for PMS. The black line was obtained by mixing the cytochrome with buffer, to check if the protein was in the fully reduced state. Residual dissolved oxygen in the buffer was responsible for the slight oxidation of OmcA by the buffer.

The oxidation of the outer membrane cytochromes OmcA, MtrC, MtrF, and UndA by AQDS, RF, FMN, and PMS is presented in Figure 2. PMS oxidized all the cytochromes at a rate too fast to be measured by stopped-flow, and only the fully oxidized state was observed for the proteins. The other electron shuttles AQDS, FMN, and RF are incapable of fully oxidizing the cytochromes. The outer membrane cytochromes OmcA and MtrC settle at approximately 0.4 of reduced fractionafter reacting with AQDS, and after reducing RF and FMN they reach approximately 0.5 of reduced fraction. MtrF settles at approximately 0.6 of reduced fraction after reacting with AQDS, RF, and FMN, while UndA achieves approximately 0.5 of reduced fraction with these three electron shuttles.

All experiments were performed with enough excess of electron shuttles to allow the full oxidation of the proteins. The fact that for all electron shuttles but PMS this oxidation is incomplete shows that a thermodynamic equilibrium has been reached. In fact, the reduction potential of the PMS is more positive than of the other electron shuttles and therefore capable of fully draining the electrons out of the cytochromes. Moreover, the analogous behavior observed for the proteins with RF and FMN are in agreement with the similar reduction potential of these flavins (Table 2).

The reactions of AQDS, FMN, RF, and PMS with the outer membrane cytochromes were too fast to establish the kinetic rates and for most cases reached the endpoint within the deadtime of the apparatus (3 ms measured according to the manufacturer's instructions). Fitting the data with a single exponential establish 10^2^ s^−1^ as the lower limit of the overall observed electron transfer rate constant between OmcA, MtrC, and UndA and the various electron shuttles. However, the kinetic behavior of the different proteins is manifestly distinct. As seen in Figure 2, the oxidation of OmcA by FMN and RF is faster than the other three proteins. Among all the cytochromes, MtrF reacts more slowly with flavins and AQDS, taking approximately 30 s to stabilize at approximately 0.6 of reduced fraction. The oxidation of OmcA, UndA, and MtrC by AQDS occurred at faster rates and are similar to each other.

The different endpoints observed for the proteins with the same electron shuttle correlate with the redox properties of the different proteins. Potentiometric titrations monitored by UV-Vis absorption spectroscopy revealed that the hemes of MtrC and OmcA are reduced over a similar potential window spanning approximately 400 mV, from fully oxidized (0 mV) to fully reduced (−400 mV), but display distinguishable shapes (Hartshorne et al., 2007; Bodemer and Antholine, 2010). MtrF titrates at slightly more positive potentials (from 50 to −400 mV) (Richardson et al., 2012). These data agree with the observation that MtrF becomes less oxidized after reacting with AQDS and flavins, and does not achieve a fully oxidized state after reacting with PMS.

### Nuclear magnetic resonance experiments

#### ^1^H NMR experiments

The outer membrane cytochromes MtrC, OmcA, and MtrF contain *c*-type hemes with bis-histidinyl coordination, which are diamagnetic in the reduced state and low-spin paramagnetic in the oxidized state. In the oxidized state, the methyl substituents at the periphery of the heme appear in the high frequency region of the NMR spectra above 10 ppm, well resolved from the protein envelope. This makes them convenient targets for monitoring of interactions taking place in the proximity of the hemes and therefore relevant for electron transfer. The perturbation of the heme signals by the electron shuttles was used to investigate the interaction of AQDS, FMN, RF, and PMS with OmcA, MtrC, MtrF, and UndA.

**Figure 3:**
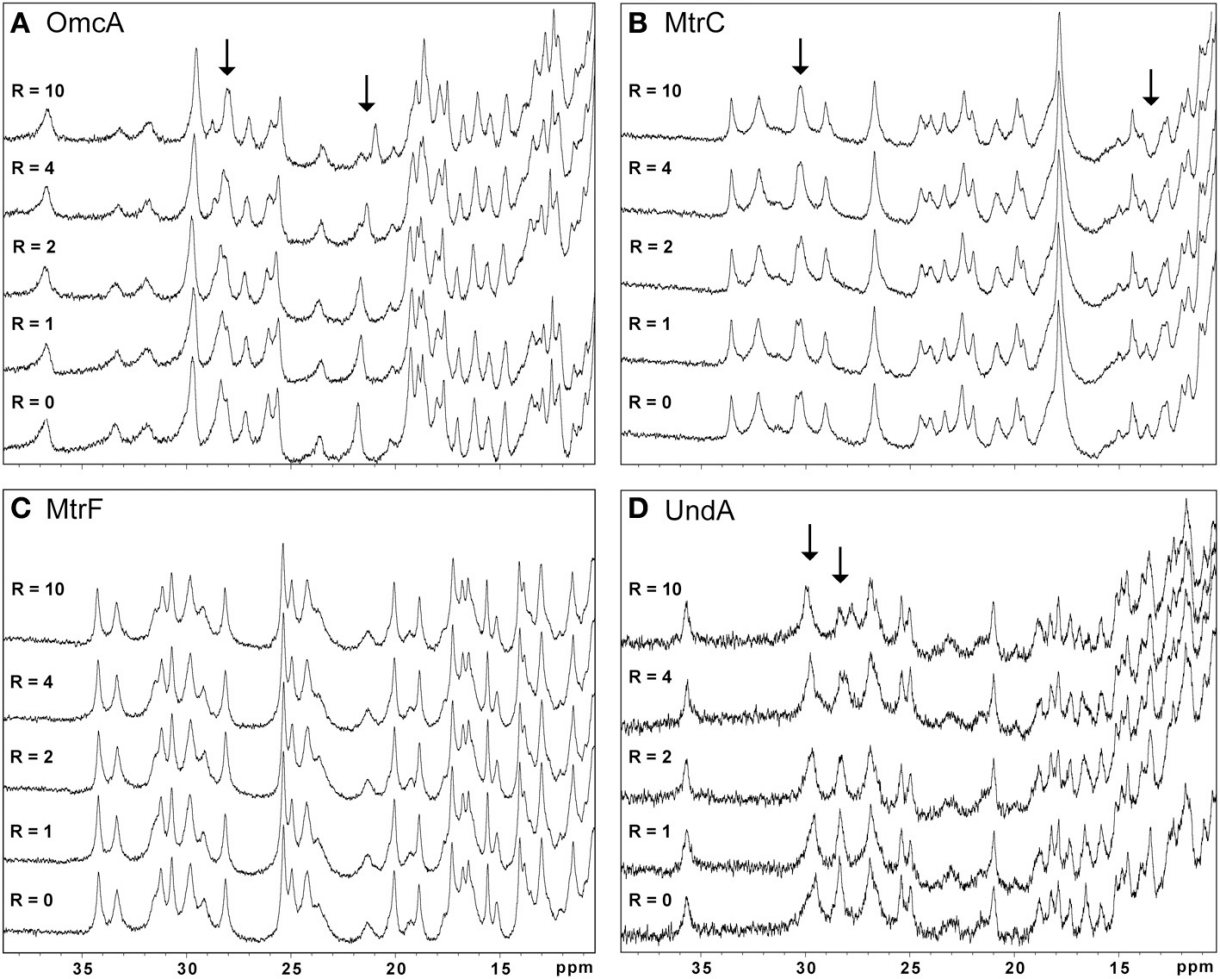
**High frequency region of the ^1^H-1D NMR spectra of OmcA (A), MtrC (B), MtrF (C), and UndA (D) in the presence of increasing amounts of AQDS**. This region is populated by the heme methyl signals. The *R*-value corresponds to the molar ratio of [AQDS]/[Cyt].

Figure 3 shows that the addition of AQDS promoted noticeable spectral changes in OmcA, MtrC, and UndA while the NMR spectra of MtrF remained unchanged. Binding reversibility between the target multiheme cytochromes and the electron shuttles was confirmed by comparing spectra recorded before the experiments with spectra recorded after the removal of the electron shuttle.

The other electron shuttles RF, FMN, and PMS, only promoted significant changes in the heme signals of OmcA and MtrC indicating that an intimate interaction between the electron shuttle and the protein in the vicinity of the hemes only occurs in these two cytochromes (data not shown). However, the lack of the three-dimensional structures of these proteins precludes the determination of the interaction site.

Due to the broadness of the signals and crowded nature of the ^1^H NMR spectra of deca- and undeca-heme cytochromes, these data are not suitable for a confident determination of binding affinities and stoichiometries of the ligands (Figure S1). To obtain this detailed mechanistic information FMN was selected as a representative ligand. This electron shuttle is unique among those tested in this work in containing a single phosphorus nucleus that can be easily followed by ^31^P NMR experiments, enabling the monitoring of FMN binding to the various target proteins.

#### ^31^P NMR experiments

**Figure 4:**
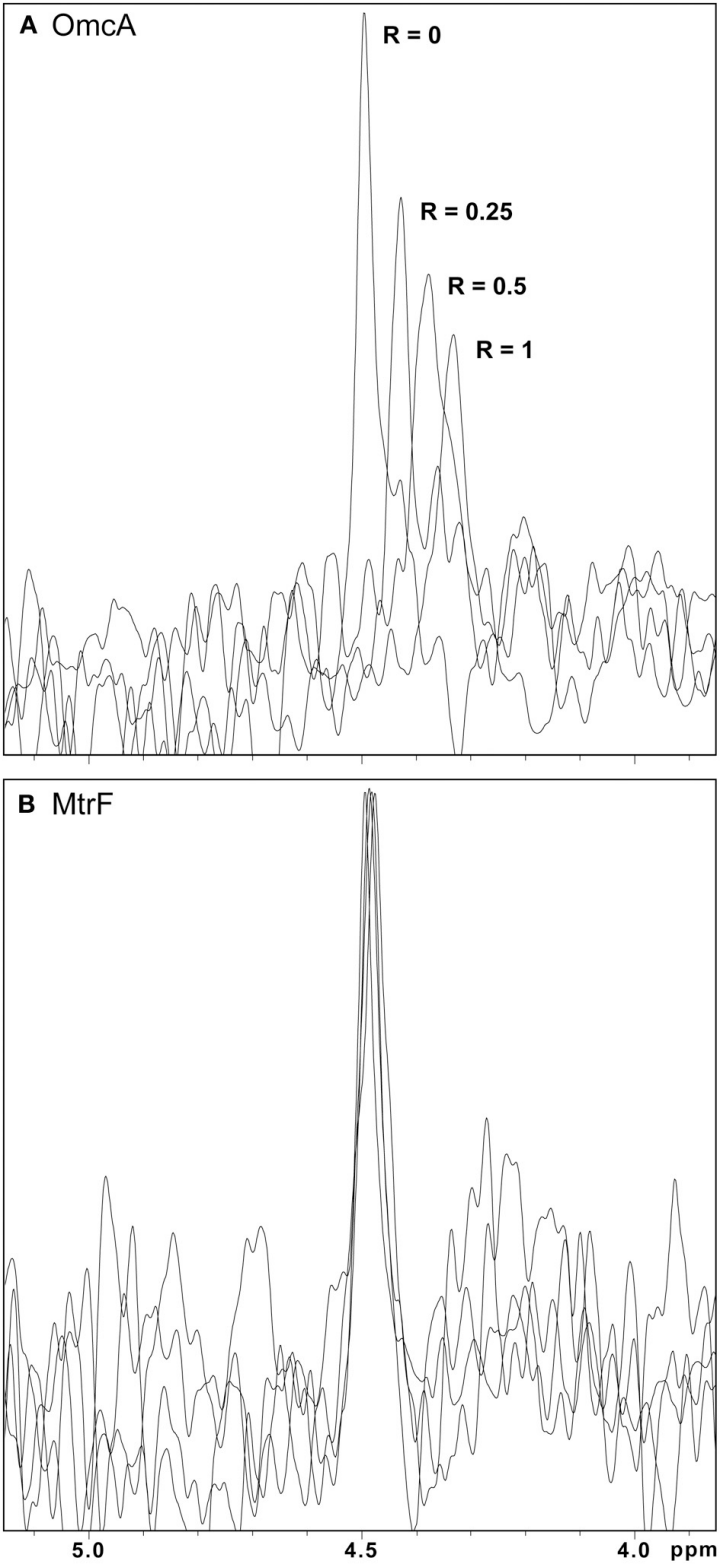
**^31^P-1D NMR spectra of FMN in the presence of increasing amounts of OmcA and MtrF. (A)** Spectral changes of the signal with increasing amounts of OmcA, indicating interaction with FMN. **(B)** The lack of significant changes in the signal of FMN with increasing amounts of MtrF indicates the absence of interaction with FMN. In both spectra the *R*-value corresponds to the molar ratio of [Cyt]/[FMN].

**Figure 5:**
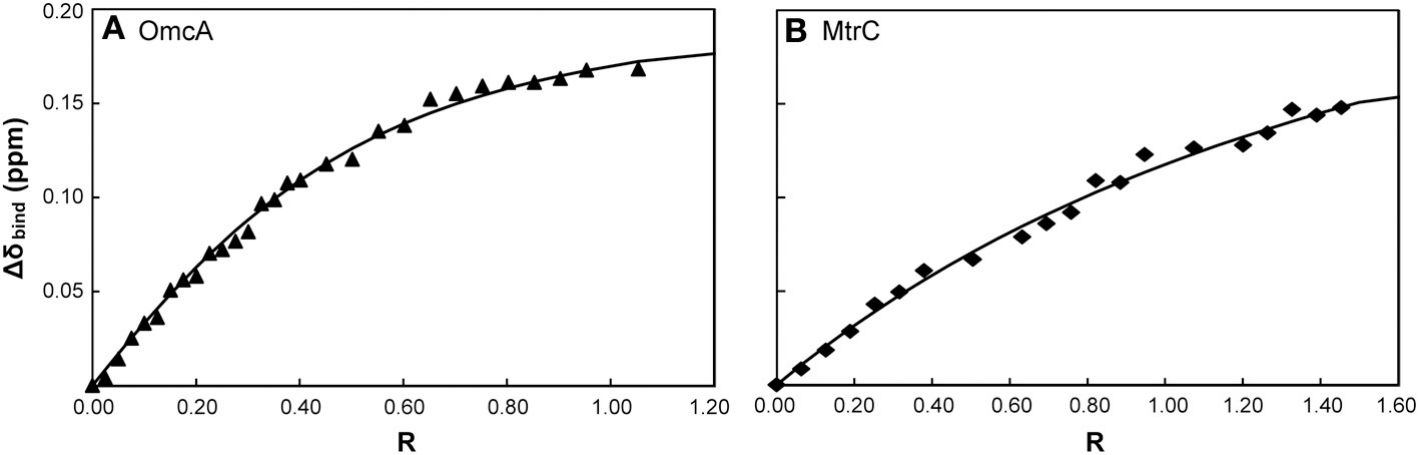
**Binding curves of FMN with the outer membrane cytochromes OmcA (A) and MtrC (B) monitored by ^31^P-1D NMR**. The chemical shift perturbation of the signal of the phosphorous nucleus of FMN are plotted as a function of the molar ratio of [Cyt]/[FMN]. The solid lines represent the best global fit to the binding model.

The molecular insights on the interaction between the outer membrane cytochromes and FMN were studied with ^31^P NMR experiments to follow the signal of FMN with increasing amounts of protein. As no other phosphorous is found in the protein or in the electron shuttle, the NMR spectra contain only the signal of FMN and of the phosphate buffer. NMR spectroscopy is exquisitely sensitive to changes in the chemical environment, and perturbations of the FMN signal indicate an interaction with the protein. Figure 4 illustrates this phenomenon by presenting changes of the FMN signal in the presence of increasing amounts of the outer membrane cytochromes. Only increasing amounts of OmcA and MtrC lead to significant changes on the chemical shifts of the FMN signal (Figures 4, 5). These changes reveal that ligand binding to these proteins is transient and occurs in the fast regime on the NMR timescale.

The macroscopic dissociation constant values and the number of binding sites obtained for both OmcA and MtrC are presented in Table 3. The values of the apparent dissociation constants are typical of redox interactions and indicate that these proteins have a weak affinity for FMN. This allows for the rapid turnover of the ligand, as expected for an electron shuttling mechanism. The number of FMN binding sites in OmcA is two, while MtrC has only one binding site.

**Table 3:** **Dissociation constants and stoichiometry of binding for FMN with OmcA and MtrC**.

	***n***	**β_*d*_ (μM)**
OmcA	2 (0.1)	29 (11)
MtrC	1 (0.2)	255 (126)
MtrF	–	–
UndA	–	–

*These values were calculated as described in the Materials and Methods section. Standard errors are shown in parenthesis and were calculated from the diagonal elements of the covariance matrix*.

### Molecular docking simulation

**Figure 6:**
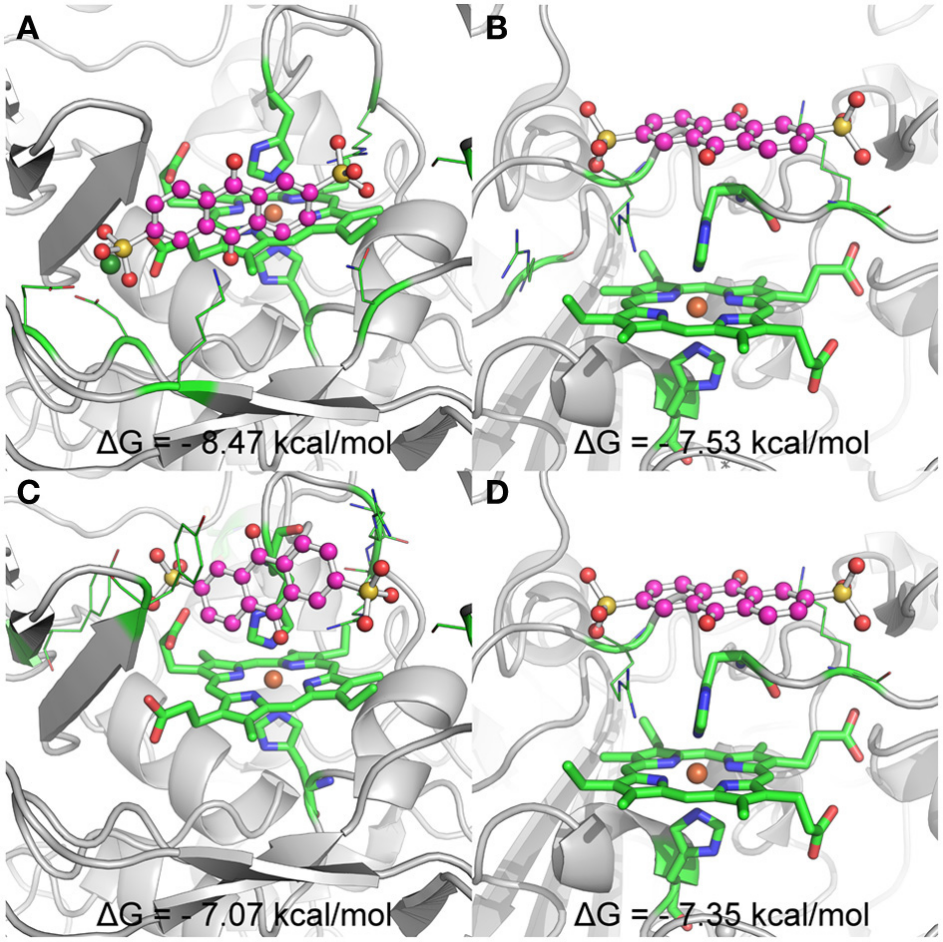
**Representation of the binding conformations of AQDS close to heme groups II and VII of UndA, and respective estimated binding free energies**. The heme groups and the histidines coordinating the iron atom are represented in sticks and the residues involved in key interactions with AQDS are represented in lines. The color code for these groups is: green for carbon, blue for nitrogen and red for oxygen. Iron is represented as a dark orange sphere. AQDS is shown in “ball and sticks” representation. The rest of the protein is represented as gray cartoon for clarity. **(A,B)** show the binding conformations with lower energy close to hemes II and VII, respectively, for the docking experiments including the Ca ion (dark green sphere) close to heme II. **(C,D)** show the binding conformations with lower energy close to hemes II and VII, respectively, for the docking experiments where no free ions where considered.

Up to date, three dimensional structures are only available for the outer membrane cytochromes MtrF and UndA. Given that only the interactions of AQDS with UndA gave rise to significant changes in the methyl proton signals in the NMR spectra, this protein-ligand complex was chosen to perform molecular docking. When the Ca^2+^ cation was included in the protein, the most populated clusters of solutions contained conformations where the AQDS molecule is found in the close vicinity of hemes II and VII (Figure 6). These two docking solutions, represented in Figures 6A,B are separated by an estimated binding free energy difference of ~1 kcal.mol^−1^, suggesting that heme II will be the preferential target of AQDS. The binding site near heme II is highly favored due to the electrostatic interaction between the negatively charged AQDS (in the sulfonate groups) and the Ca^2+^ cation. If the Ca^2+^ cation is removed and docking is performed, this result is inverted, with the docking solution near heme II (Figure 6C), being 0.4 kcal.mol^−1^ higher in energy (and different from the one observed in Figure 6A) than the docking solution near heme VII; therefore, under these conditions, the docking of AQDS would be slightly more favored near heme VII. Nevertheless, as referred before, it is likely that the Ca^2+^ binding site is physiological, suggesting that heme II will be the binding site of AQDS in this protein. But, clearly, it will be either in hemes II and VII.

## Discussion

The concept of mediated electron transfer between DMRB and metallic solids is well established for some time (Newman and Kolter, 2000). Mediated electron transfer in *Shewanella* was found to be the primary mechanism of extracellular electron transfer (Kotloski and Gralnick, 2013). Flavins, such as FMN and RF, were identified as the major endogenous secreted electron shuttles by *Shewanella* sp. (Marsili et al., 2008; Jiang et al., 2010), but other electron shuttles have been documented to facilitate the reduction of insoluble compounds (Hernandez et al., 2004; Lovley, 2006). The reduction of electron shuttles in *Shewanella* is dependent on multiheme cytochromes, some of them positioned at the cell surface (Ross et al., 2009; Bücking et al., 2010; Coursolle et al., 2010). The determination of the crystal structure of the outer membrane decaheme cytochrome MtrF from *Shewanella oneidensis* MR-1 and undecaheme cytochrome UndA from *Shewanella* sp. HRCR-6, lead to the suggestion that all members of this outer membrane cytochrome family share a similar structure of a four domains fold that consists of two multiheme domains and two flanking β-barrel domains. The kinetics of oxidation showed that among the outer membrane cytochromes, OmcA and MtrC are equally competent in the reduction of the electron shuttles, reducing flavins at a faster rate than their homologs. The importance of these two proteins in mediated electron transfer is in agreement with the strong growth defect under dissimilatory metal-reducing conditions observed in *Shewanella* mutants lacking them (Coursolle et al., 2010). The kinetic behavior observed for UndA is very similar to that of OmcA and MtrC, suggesting that this protein may operate in a similar manner in the outer membrane of *Shewanella* sp. HRCR-6.

NMR spectroscopy is a powerful technique to study protein-protein, and protein-ligand interactions (Worrall et al., 2003; Fonseca et al., 2013; Dantas et al., 2014). This work reveals that the interactions between the electron shuttles tested and OmcA and MtrC occur close to the hemes. These results suggest that these proteins may operate as extracellular redox hubs capable of interacting with numerous electron shuttles to optimize the respiratory flexibility of *Shewanella*.

UndA, although kinetically similar to OmcA and MtrC, only exhibited clear changes in the NMR heme signals in the presence of AQDS. Molecular docking simulations performed with UndA and AQDS showed that the interaction occurs near heme II and heme VII (but most probably near heme II), with the closest distance between the iron and the aromatic ring of 6.8 and 8.1 Å, respectively. This is in agreement with the proposal made on the basis of the protein structure, despite the lack of success in observing bound ligands in crystals soaked with a variety of electron shuttles (Edwards et al., 2012b). The interaction with the other electron shuttles tested in the present work must occur further away from the hemes, to account for the lack of perturbation of the heme signals in the NMR spectra, as well as the slower kinetics observed.

The NMR spectra of MtrF showed no significant disturbance of the heme signals upon addition of these redox shuttles. This suggests a more distant interaction site, in agreement with the slower rates observed kinetically. Molecular docking simulations of the interaction between MtrF and AQDS to allow for the comparison with the results from UndA showed only a family of docking solutions but with the ligand located more than 18 Å from the nearest heme iron (Figure S2). This suggests that, although capable of replacing MtrC in the reduction of several electron acceptors (Bücking et al., 2010; Coursolle and Gralnick, 2010, 2012), MtrF may have a distinct function from its outer membrane homologs. Indeed, *Shewanella* mutants lacking this cytochrome were found to have distinct growth deficiencies when compared with mutant lacking its homologs (Gao et al., 2010). MtrF either evolved to interact with a different class of ligands, structurally different from these electron shuttles, or is not designed to interact with soluble electron shuttles. This agrees with the lack of success in observing bound FMN in MtrF crystals soaked with this electron shuttle (Clarke et al., 2011).

**Figure 7:**
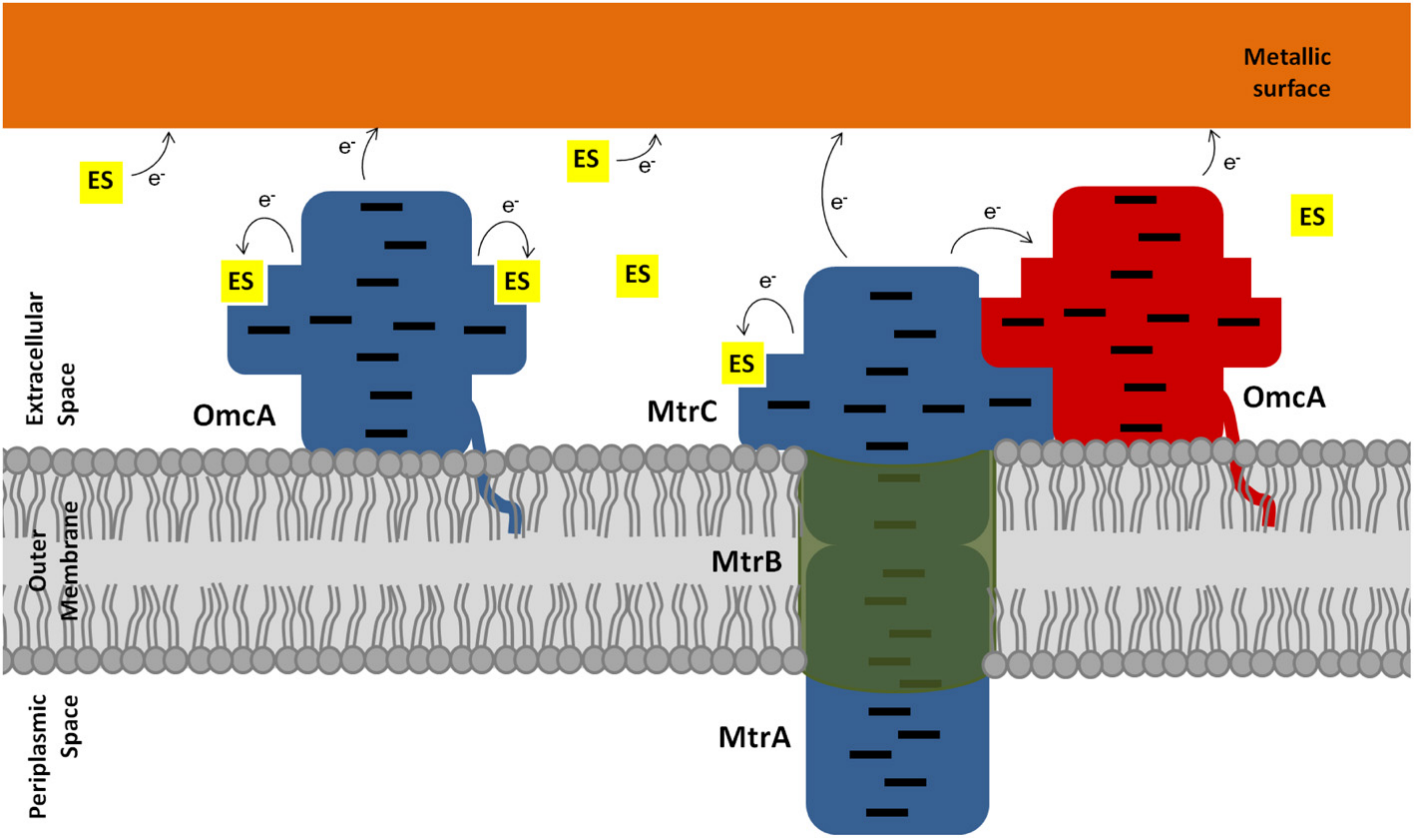
**Schematic representation of the extracellular electron transfer pathway of *Shewanella***. Electrons, represented as e^−^, can flow from the outer membrane cytochromes directly or indirectly to metallic surfaces. The electron shuttles (ES) are represented by small yellow squares. Blue proteins represent the reduced state, whereas red proteins represent the oxidized state. The hemes in the outer membrane cytochromes are represented as black lines.

^31^P NMR experiments enabled the determination of the dissociation constant of both OmcA and MtrC with FMN. The values are compatible with ligand binding that is physiologically relevant, given the concentration range of flavins found in the extracellular medium of *Shewanella* (Marsili et al., 2008; Von Canstein et al., 2008; Ross et al., 2009). Despite the similar kinetic behavior, OmcA and MtrC appear to have different ligand binding stoichiometries and distinct affinities for FMN, which may be related with functional specificity of these proteins. While OmcA can bind two FMN molecules, MtrC only binds one. These results, together with the reports in the literature of variable stoichiometry of 1:1 and 2:1 for the OmcA:MtrC complex (Ross et al., 2007; Zhang et al., 2009) and antibody functionalized AFM data showing a different distribution pattern for MtrC and OmcA at the surface of *Shewanella* (Lower et al., 2009), support the scenario proposed in Figure 7. MtrC is inserted in the β-barrel porin of the MtrCAB-OmcA complex and can receive electrons from the cell metabolism. It can transfer them to its redox partner OmcA, to soluble electron shuttles or directly to solid extracellular acceptors. Reduced OmcA can move by lateral diffusion in the cell surface of *Shewanella* attached only by the lipidated cysteine at the N-terminus. This mechanism would allow OmcA to move in the surface of *Shewanella*, receiving electrons from MtrC and transferring them to electron shuttles, contributing both to the discharge of the electrons from the metabolism and also for modulating the charge of cell surface patches for control of adhesion to other cells or surfaces. Given that MtrC and OmcA can charge up to ten electrons, simple electrostatics can account for reduced MtrC to be attractive to oxidized OmcA and repulsive to reduced OmcA allowing the dissociation of OmcA from the complex. This scenario is also supported by the report of larger number of copies of OmcA than MtrC at the surface of SOMR1 (Ross et al., 2009) and thereby each copy of MtrC could charge more than one copy of OmcA. Exploration of this hypothesis however requires the structural characterization of these two proteins or eventually the full MtrCAB-OmcA complex. Nonetheless, the structure of UndA and the families of docking solutions with AQDS can provide some hints, given the homology within this family of proteins. Docking solutions for AQDS with UndA show the ligand inserted on hemes II and VII from the side of heme XI (Figure S3). This supports a proposal that the most likely orientation of UndA in the surface of *Shewanella* sp. strain HRCR-6 is with heme V close to the membrane and heme XI pointing outwards. This proposal is independently supported by the observation of an aminoacid sequence closely related to the consensus sequence for hematite binding located in the vicinity of hemes X and XI (Lower et al., 2008). Site directed mutagenesis can now proceed to mutate residues in the vicinity of the AQDS binding sites to modulate the activity of this protein.

The data reported in this work show that all outer membrane cytochromes from *Shewanella* are oxidized by flavins, phenazines, and humic acid analogs. This may be another manifestation of the versatility and resilience of the electron transfer chain of this organism that maintains multiple alternative paths. However, not all cytochromes appear to have interactions with these ligands in the close proximity of the hemes, and those with affinities that are physiologically relevant given the typical concentrations of redox shuttles in the environment show different stoichiometries. This shows that the road ahead for manipulation of the extracellular electron transfer activity of *Shewanella* as a model DMRB organism may take multiple paths. Of the two proteins for which high resolution structural information is available, the lack of kinetic and docking evidence for close interaction between redox shuttles and the hemes of MtrF in comparison with the results obtained for UndA make the later a more interesting target for these studies. Furthermore, given the homology between UndA and OmcA, UndA may contribute both to surface adhesion and extracellular electron transfer.

### Conflict of interest statement

The authors declare that the research was conducted in the absence of any commercial or financial relationships that could be construed as a potential conflict of interest.

## Supplementary material

### Addendum

While this manuscript was being evaluated, the structure of OmcA became available on the Protein Data Bank (4LMH) [Edwards MJ et al. FEBS Letts 2014, 588: 1886–1890]. NMR data obtained upon docking all four redox shuttles with OmcA revealed that in all cases docking disturbed the heme signals and therefore was relevant for electron transfer. Furthermore, it revealed that the spectral changes in the heme signals were different for different ligands, suggesting different binding locations (Figure S4). Given these experimental observations and the availability of the structure of OmcA, we took the opportunity to perform docking simulations of these ligands on the structure of OmcA. These simulations followed the same procedures indicated in the Materials and Methods section for the interaction of AQDS with UndA. As observed in the structure of UndA, OmcA also contains a calcium atom near heme II even though calcium was not used in the crystallization buffer. This reinforces the proposal already made in the main text that this calcium atom is physiologically relevant. The results of the docking simulations reveal that the various redox shuttles display different preferences for locations in the surface of OmcA near the hemes. For the shuttles with negative charges, AQDS and FMN, the cluster of solutions with lower energy shows docking near heme II, with the shortest distances between the iron and the aromatic rings of 6.8 and 7.2 Å, respectively (Figures S5, S6). For neutral and positively charged ligands the scenario is different. Riboflavin displays the cluster of solutions with lowest energy near hemes IX and X, with the shortest distances between the irons and the ring of 5.8 and 10.7 Å, respectively (Figure S7). For the positively charged phenazine the family of solutions with lowest energy is found near to heme X but in a different location to that preferred by riboflavin (Figure S8). In this case the closest distance between the iron and the ring is 14.2 Å. Overall these data suggest a functional specificity for each of the hemes in these decaheme cytochromes in their interactions with soluble redox shuttles, and a physiological role for the calcium atom near heme II. These results reinforce the conclusions on the main text by bringing OmcA to the fore as an additional suitable target for the rational manipulation of the extracellular electron transfer activity of *Shewanella* as a model DMRB.
