# Supplementary material for: Exploring the molecular mechanisms of electron shuttling across the microbe/metal space
Source: Front Microbiol. 2014 Jun 27;5:318. doi: 10.3389/fmicb.2014.00318 (PMC4073285; doi:10.3389/fmicb.2014.00318)
Supplement: Supplementary file 1 [file DataSheet1.DOCX]

***Supplementary Material***

**Exploring the molecular mechanisms of electron shuttling across the microbe/metal space**

**Catarina M. Paquete^1^, Bruno M. Fonseca^1^, Davide R. Cruz^1^, Tiago Pereira^1^, Isabel Pacheco^1^, Cláudio M. Soares^1^, Ricardo O. Louro^1^***

^1^Instituto de Tecnologia Química e Biológica António Xavier- Universidade Nova de Lisboa, Av. da República - EAN, 2780-157 Oeiras, Portugal

*** Correspondence:** Ricardo O. Louro, Instituto de Tecnologia Química e Biológica António Xavier, Universidade Nova de Lisboa, Av. da República - EAN, 2780-157 Oeiras, Portugal.

louro@itqb.unl.pt

1. **Supplementary Figures and Tables**

## Suplementary Figures





Supplementary Figure 1. High frequency region of the ^1^H-1D NMR spectra of FMN in the absence and in the presence of increasing amounts of OmcA. The R value corresponds to the molar ratio of [OmcA]/[FMN].


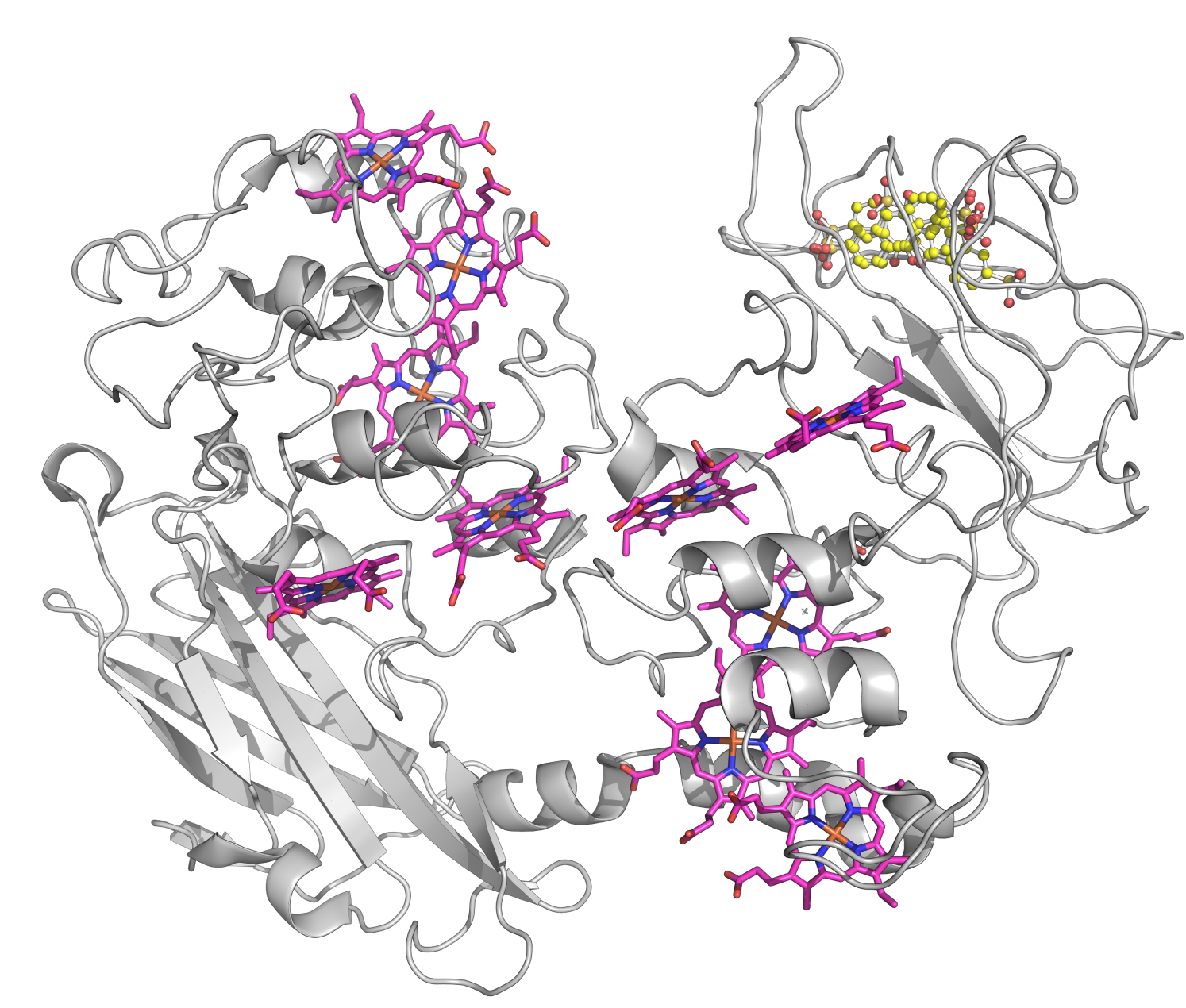


Supplementary Figure 2. Schematic representation of the predicted binding locations of AQDS on the surface of MtrF. The AQDS conformations shown in the image represent the most populated clusters with lower energy. The protein is shown as gray cartoon, the heme groups are shown in sticks and the ligand is shown in ball and stick representation. To distinguish the AQDS from the heme groups, the carbons of the former are colored in yellow, while the ones from the latter are colored in magenta. AQDS is positioned close to the heme group II of MtrF.


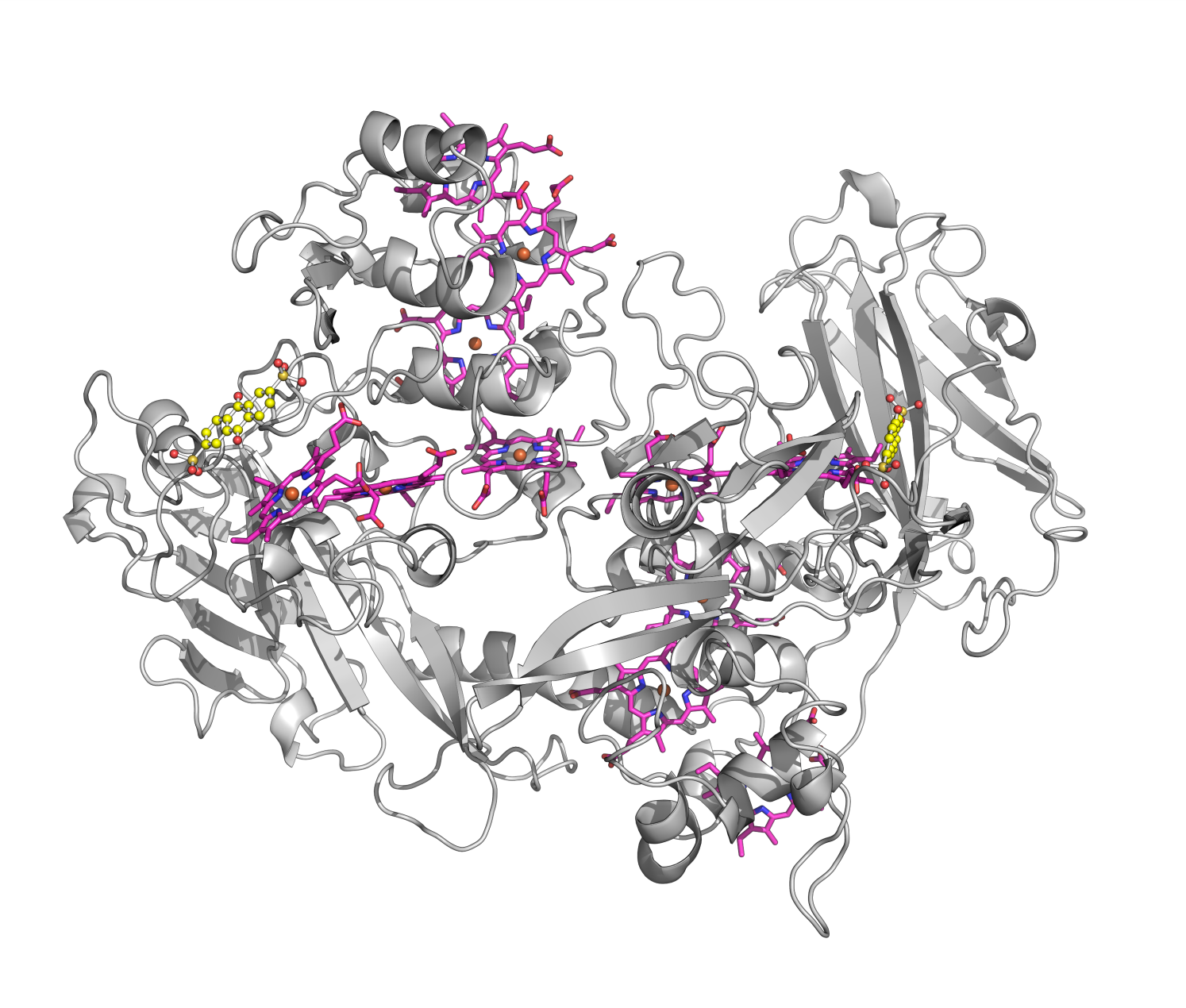


Supplementary Figure 3. Schematic representation of the two lower energy conformations predicted by docking of AQDS on the surface of UndA. The AQDS conformations shown in the image represent the most populated clusters with lower energy. The protein is shown as gray cartoon, the heme groups are shown in sticks and the ligand is shown in ball and stick representation. To distinguish the AQDS from the heme groups, the carbons of the former are colored in yellow, while the ones from the latter are colored in magenta The binding of AQDS occurs in the vicinity of the heme groups II (at the right hand side of the figure) and VII (at the left hand side of the figure) of UndA.


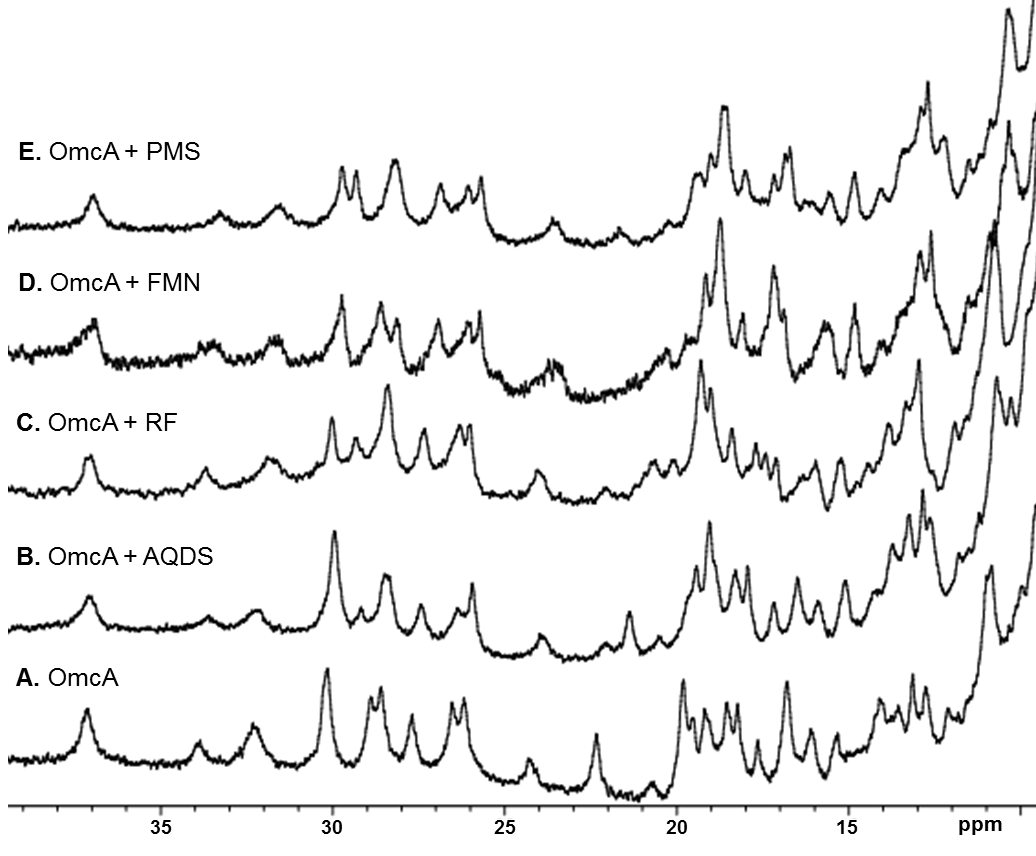


**Supplementary Figure 4**. **High frequency region of the ^1^H-1D NMR spectra of OmcA.** (A), spectrum of the pure OmcA, (B) spectrum with excess AQDS, (C) spectrum with excess riboflavin, (D) spectrum with excess FMN, and (E) spectrum with excess phenazine. Spectra B to E are different from A and also among themselves.


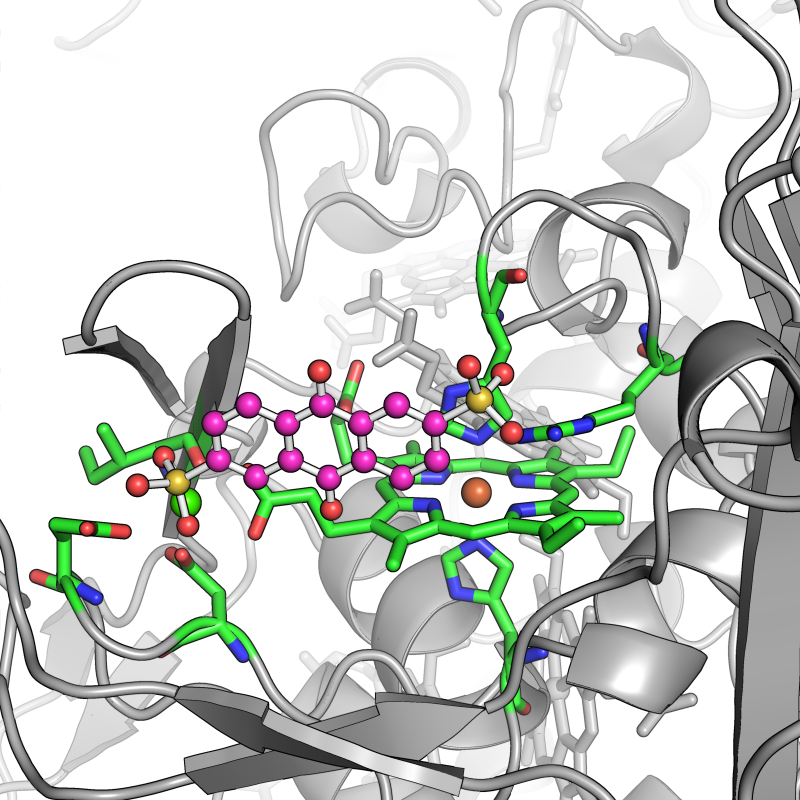


**Supplementary Figure 5. Schematic representation of the lowest energy conformation predicted by docking of AQDS on the surface of OmcA.** The protein is shown as gray cartoon, the heme groups are shown in sticks and the ligand is shown in ball and stick representation. The carbon atoms of the heme II group, the histidine side chains coordinating the iron atom and the residues coordinating the Ca(II) ion are shown in green. The carbon atoms of AQDS are shown in magenta.

**
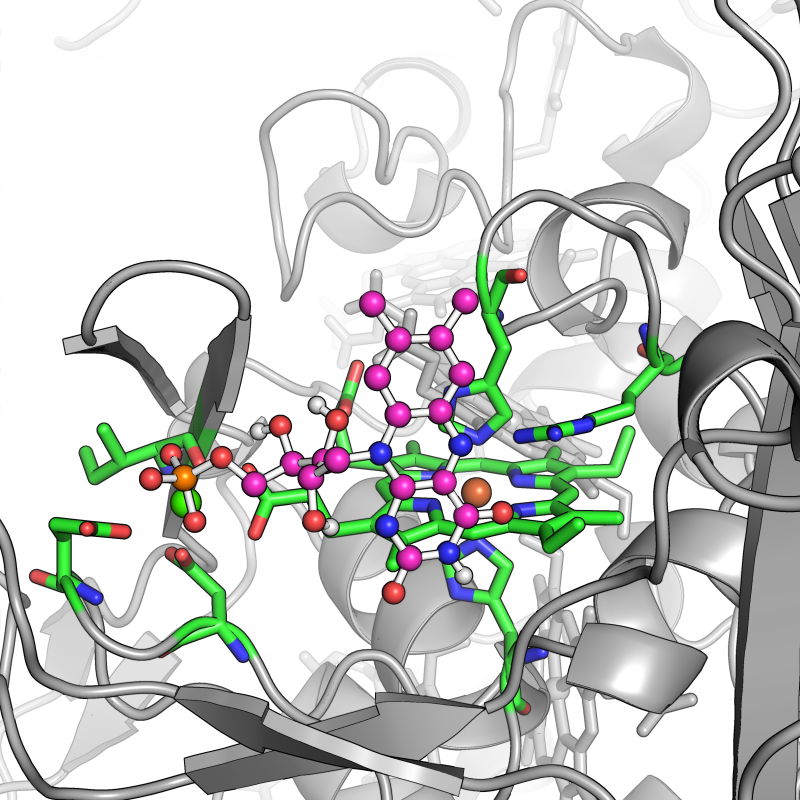
**

**Supplementary Figure 6. Schematic representation of the lowest energy conformation predicted by docking of FMN on the surface of OmcA.** The protein is shown as gray cartoon, the heme groups are shown in sticks and the ligand is shown in ball and stick representation. The carbon atoms of the heme II group, the histidine side chains coordinating the iron atom and the residues coordinating the Ca(II) ion are shown in green. The carbon atoms of FMN are shown in magenta.

**
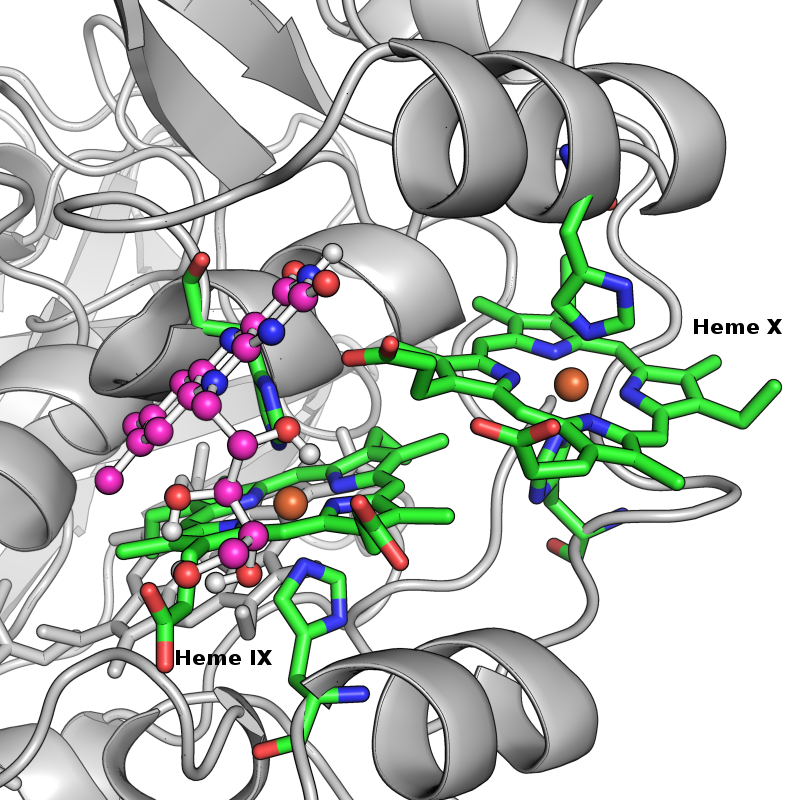
**

**Supplementary Figure 7. Schematic representation of the lowest energy conformation predicted by docking of riboflavin on the surface of OmcA.** The protein is shown as gray cartoon, the heme groups are show in sticks and the ligand is shown in ball and stick representation. The carbon atoms of hemes IX and X, and the histidine side chains coordinating the iron atom are colored in green. The carbon atoms of riboflavin are shown in magenta. Labels were introduced to distinguish both heme groups.

**
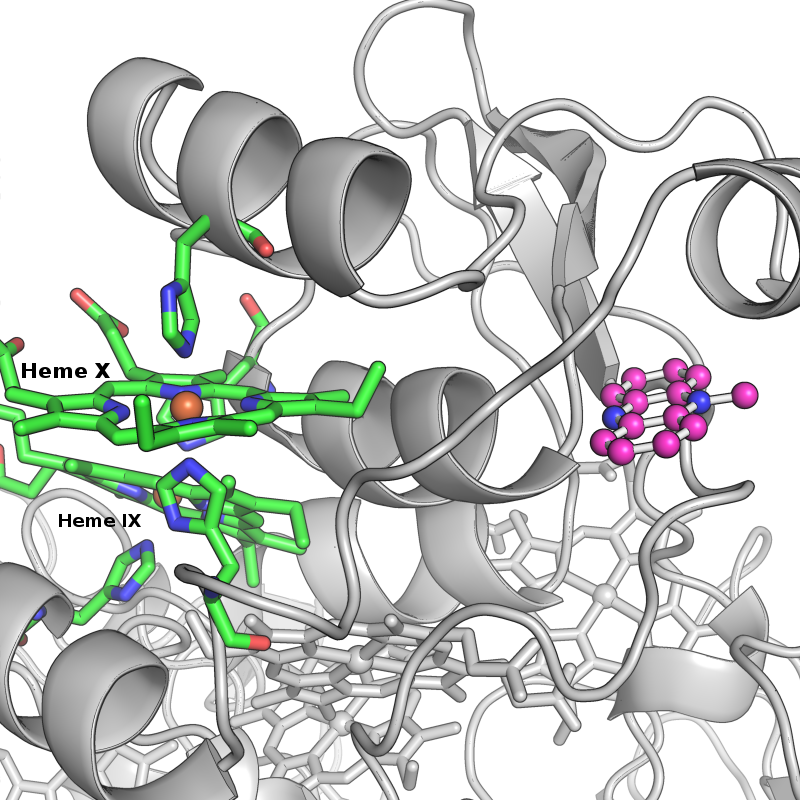
**

**Supplementary Figure 8. Schematic representation of the lowest energy conformation predicted by docking of phenazine on the surface of OmcA.** The protein is shown as gray cartoon, the heme groups are shown in sticks and the ligand is shown in ball and stick representation. The carbon atoms of hemes IX and X, and the histidine side chains coordinating the iron atom are colored in green. The carbon atoms of phenazine are shown in magenta. Labels were introduced to distinguish both heme groups.
